# Patch Area and Soil Resource Availability Outweigh Heterogeneity in Shaping Karst Plant Diversity During Early Restoration

**DOI:** 10.1002/ece3.72417

**Published:** 2025-10-30

**Authors:** Weixue Luo, Ying Lei, Yijie Zhao, Wenqi Yang, Haohan Du, Jie Luo, Xuman Guo, Wenjing Tao, Zongfeng Li, Jianping Tao, Jinchun Liu

**Affiliations:** ^1^ Key Laboratory of Eco‐Environments in Three Gorges Reservoir Region (Ministry of Education), Chongqing Key Laboratory of Plant Ecology and Resources Research in Three Gorges Reservoir Region, School of Life Sciences Southwest University Chongqing China; ^2^ Chongqing Jinfo Mountain Karst Ecosystem National Observation and Research Station Southwest University Chongqing China

**Keywords:** limestone, patch area, phylogenetic diversity, soil depth, species richness, vegetation restoration

## Abstract

Karst landscapes, vital to global terrestrial ecosystems, are characterized by fragmented soil patches with low resource availability and high resource heterogeneity. However, the relative roles of patch properties, soil resource availability, and heterogeneity in shaping plant species and phylogenetic diversity remain unclear. To address this, we conducted in situ observations across 27 10 m × 10 m plots in three karst abandoned farmlands to quantify the effects of patch properties, soil resource availability, and heterogeneity on plant species composition, species diversity, and phylogenetic diversity during early restoration. Patch area strongly influenced species richness (*R*), Shannon–Wiener index (*H*′), phylogenetic diversity (*PD*), and net relatedness index (*NRI*). Soil resource availability, notably soil depth and water content, consistently enhanced *R*, *H*′, and *PD*. Conversely, soil resource heterogeneity had minimal impact on species diversity but significantly shaped *NRI* and nearest taxon index (*NTI*). Deterministic and stochastic processes jointly drove the community assembly of early herbaceous communities in karst abandoned farmland. These findings highlight that patch area and soil resource availability are primary drivers of plant diversity in karst restoration, offering a patch‐scale framework to guide biodiversity conservation and vegetation recovery in degraded karst ecosystems.

## Graphical abstract

Patch area strongly influenced species and phylogenetic diversity in karst areas. Soil resource availability increased species and phylogenetic diversity. Soil nutrient heterogeneity shaped phylogenetic structure in early karst restoration. Patch shape and soil resource quantity outweighed heterogeneity in karst diversity. Deterministic and stochastic processes shaped early herbaceous community assembly.

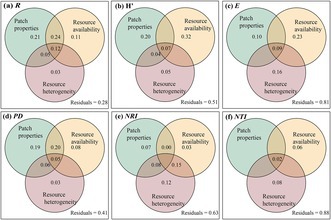

## 1 Introduction

Karst landscapes, shaped by unique geological and socio‐economic pressures, have experienced increasing degradation by rocky desertification, resulting in severe ecological challenges, such as soil erosion, water loss, and vegetation degradation (D'Ettorre et al. 2024; Li et al. 2024). In recent years, abandoned lands in karst regions have been expanding due to intensified soil erosion and urban–rural development, such as land conversion and infrastructure (Green et al. 2019; Zhang et al. 2023), exacerbating karst rocky desertification. Natural restoration of abandoned land is widely recognized as one of the most effective strategies to mitigate rocky desertification (Lu et al. 2023; Zhao, Pereira, et al. 2020). Notably, the early stage of vegetation restoration plays a pivotal role in shaping the long‐term dynamics of plant community succession (Veen et al. 2018). Therefore, understanding the patterns and drivers of plant diversity during early restoration is essential for both vegetation recovery and biodiversity conservation.

Karst ecosystems are characterized by fragmented soil patches with low soil resource availability and high soil resource heterogeneity, driven by discontinuous soil cover and exposed limestone (Guo et al. 2024; Li et al. 2022). On the one hand, variations in the area, shape, and resource distribution of soil patches alter soil water and nutrient availability, thereby driving differences in plant community structure, species diversity, and phylogenetic diversity across soil patches (Geekiyanage et al. 2019; Liu et al. 2021; Podzikowski et al. 2025). These differences are primarily driven by soil patch properties, including patch area, isolation, perimeter, and fractal dimension (Hao et al. 2016; Hargis et al. 1998; Outhwaite et al. 2022). On the other hand, due to adverse conditions such as exposed rocks, fragmented terrain, and severe soil erosion, low soil resource availability and high soil resource heterogeneity have become two core characteristics in the karst habitats, shaping community assembly and diversity (Liu et al. 2021; Nie et al. 2012). Therefore, soil patch properties, soil resource availability, and resource heterogeneity drive plant diversity during the early restoration stage of abandoned lands in rocky desertification areas.

Soil patch properties, such as patch area, isolation, perimeter, and fractal dimension, can significantly influence biodiversity (Hargis et al. 1998). Most previous studies have emphasized the impact of patch area, demonstrating that larger patch areas typically support higher species diversity by providing greater resource pools and habitat stability (Chase et al. 2020; Ramírez‐Delgado et al. 2022). However, some studies have posited that patch shape (e.g., fractal dimension) is more indicative of species‐richness patterns than patch size (Santana et al. 2021), which is attributed to the effects of dispersal and distance to neighboring patches (Heegaard et al. 2007). In karst landscapes, larger, irregular soil patches often retain more soil and exhibit greater resource heterogeneity. Accordingly, large and complex patches have a shorter average distance to neighboring patches (of different types) than small, simple‐shaped (circular) patches have (Heegaard et al. 2007). Therefore, we support that the patch area with fractal dimension would play a vital role in determining species diversity in karst ecosystems.

As mentioned above, low soil resource availability and high soil resource heterogeneity are important selection pressures for local vegetation growth and community assembly currently and in the future in karst areas (Guo et al. 2024). Recent studies suggest that soil resource availability, including soil amount, water content, and total nitrogen content, is a key determinant of plant community structure and diversity in karst regions (Liu et al. 2020, 2021; Zhao, Shen, et al. 2020). Accordingly, low soil resource availability typically constrains plant diversity. On the other hand, moderate soil resource heterogeneity is widely recognized to promote species coexistence and enhance species diversity (Baer et al. 2020; Price et al. 2017; Questad and Foster 2008; Stein et al. 2014), but excessive heterogeneity(Lebrija‐Trejos et al. 2010), coupled with habitat fragmentation, may suppress diversity. For instance, Phoutthavong et al. (2019) found that the species diversity of ferns in karst regions with high habitat heterogeneity was lower than that in non‐karst regions with low habitat heterogeneity. Similarly, Wu et al. (2013) observed that the relationship between habitat heterogeneity and species richness shifted from positive to negative with increasing fragmentation. Therefore, we speculate that a combination of soil resource availability and soil resource heterogeneity drives plant diversity during the early restoration stage of abandoned lands, with optimal diversity arising from moderate soil resource heterogeneity and high soil resource availability.

In addition, because the properties of soil patches (e.g., patch size and shape) lead to changes in the availability and heterogeneity of soil resources (such as moisture, nitrogen, and phosphorus), soil patches often interact with soil resource availability or soil resource heterogeneity, influencing the species diversity of early plant communities in karst abandoned farmland. For example, larger patches with greater soil volume enhance resource availability (e.g., water and nutrients), facilitating niche partitioning that allows species with diverse resource requirements to coexist (Chesson 2000; Silvertown 2004). However, in fragmented karst landscapes, patch isolation can limit dispersal, thereby restricting colonization and shaping community assembly by favoring species with strong dispersal abilities (Leibold et al. 2004; Myers and Harms 2009). Conversely, smaller or more isolated patches not only have lower resource availability but also exhibit higher resource heterogeneity, which drives environmental filtering and selects for species with specific adaptations (e.g., drought tolerance), thereby reducing diversity (Cornwell and Ackerly 2009; Kraft et al. 2015).

Here, we aimed to quantify the relative contributions of patch properties, resource availability, and resource heterogeneity to species and phylogenetic diversity at the patch scale during the early restoration stage of karst abandoned lands. Furthermore, we addressed the following questions: (1) How do soil patch properties, particularly area and shape, affect species and phylogenetic diversity in the karst region? (2) What are the relative roles of soil patch properties, soil resource availability, and resource heterogeneity during the early restoration dynamics? We hypothesized that: (1) larger patch areas and more complex patch shapes (e.g., higher fractal dimensions) would significantly increase species richness, Shannon–Wiener diversity, and phylogenetic diversity, as they provide greater resource availability and diverse microhabitats (Maestre 2004); (2) soil resource availability would have a greater positive impact on species diversity than heterogeneity, while heterogeneity would more strongly influence phylogenetic structure by promoting coexistence of distantly related species through diverse microhabitats (Liu et al. 2021). Our findings would elucidate the mechanisms governing plant diversity in the early restoration stage of karst abandonment, offering valuable insights for biodiversity management and conservation efforts in karst landscapes.

## 2 Materials and Methods

### 2.1 Study Area

**FIGURE 1:**
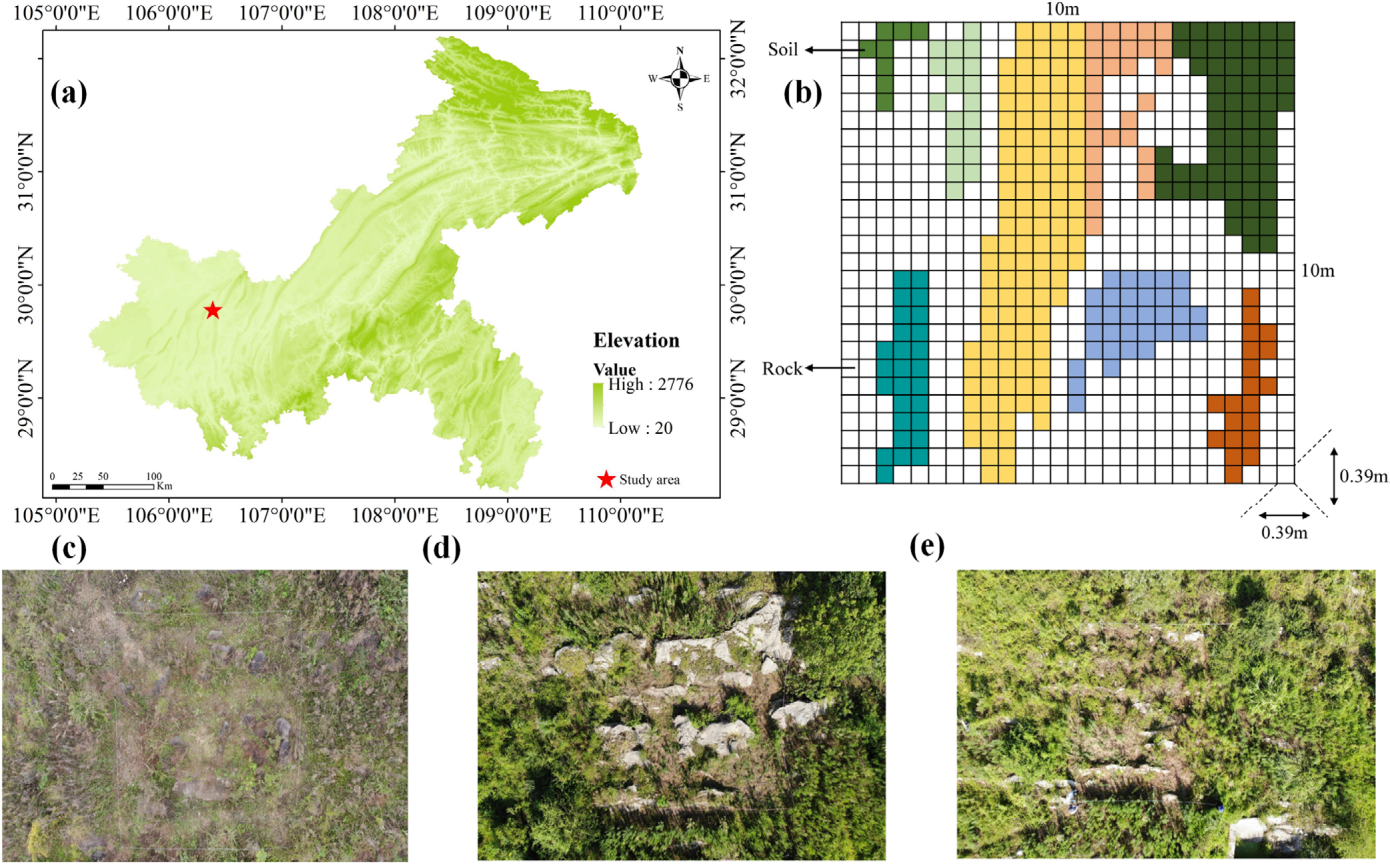
(a) Map of the study area in a karst ecosystem, with the study location marked by a red star and elevation shown in a monochromatic color scale (shades of green, ranging from 20 to 2776 m). (b) Diagram of soil patch distribution within a representative 10 m × 10 m plot (one of 27 sampled plots), where colored grids represent soil patches (with distinct colors indicating different patches) and white grids represent rocks. (c–e) Field photographs illustrating plant distribution and patch patterns across the study area.

The study was conducted in the Zhongliang Mountain, Longfeng Town, Beibei District, Chongqing, China (106°25′–106°29′ E, 29°45′–29°50′ N), a representative karst gorge landscape (Figure 1). This region has a subtropical monsoon climate with distinct seasons, abundant heat, and spatially variable precipitation. The annual average temperature is approximately 17.4°C, peaking at 40°C, and the average annual precipitation is 1078 mm, with a maximum of 1544 mm. The soil is primarily yellow calcareous and the native vegetation was evergreen broad‐leaved forest. However, due to lithology, soil erosion, and human disturbance, typical forests have been replaced by farmlands, with the main crops of corn and potato. After abandonment, dominant herbaceous species found in the area include *Conyza canadensis* , *Setaria viridis* , and *Artemisia annua* .

### 2.2 Plot Design and Data Acquisition

In August 2021, we selected three abandoned farmlands (4–6 years) with similar degrees of rocky desertification and the same land use within the study area. Each abandoned farmland was subdivided into nine 10 m × 10 m plots, totaling 27 plots, with a minimum distance of more than 20 m between adjacent plots to minimize spatial autocorrelation. Within each plot, a 26 × 26 grid was established, comprising 676 soil depth measurement points, with a distance of 0.39 m between each point (Figure 1). At each point, soil depth was measured using a steel auger. Soil patch maps were created based on the grid cells that corresponded to the soil depth measurements and actual field conditions (Figure 1). Notably, some patches appeared connected in the plan view due to varying horizontal levels caused by rocks and partial rock outcrops within the plots. Consequently, when the width of a rock was less than this distance, the patch seemed connected in the plan view. Each grid cell measured 0.39 m on each side, covering an area of 0.15 m^2^. The total number of grid cells and the area of each patch's outermost boundary were then calculated based on the soil patch maps. Additionally, each 10 m × 10 m plots were further divided into 16 subplots (2.5 m × 2.5 m), ensuring that a 1 m × 1 m quadrat can be selected in each subplot for soil sampling.

Herbaceous communities within the patches were surveyed in each plot, recording species identity, abundance, height, and coverage of each species. Soil moisture was measured at a depth of 0–10 cm using a soil multi‐parameter tester (TZS‐PHW‐4G) with a five‐point method within the quadrats. Soil samples were collected from each quadrat at a depth of 0–30 cm or until reaching the underlying rock if the soil depth was less than 30 cm. These soil samples were then individually mixed and dried in the laboratory for subsequent analyses. The total soil carbon and nitrogen content were measured using an elemental analyzer (Vario EL cube), and soil nutrient content within each patch was then calculated based on the values from all quadrats.

Across all patches, we recorded 98 plant species belonging to 84 genera and 43 families. Overall, the Asteraceae family was the most abundant, occurring in nearly all soil patches (Figure S1). In patches with an area of 15–30 m^2^, the Rosaceae family was more prevalent than the Poaceae family, while the latter dominated in other patch areas. The Leguminosae family was scarce, absent in patches ranging from 25 to 30 m^2^ (Figure S1). The important values of species (top 5) in different patches are seen in Table S1.

### 2.3 Metrics of Community Diversity and Phylogenetic Structure

#### 2.3.1 Phylogeny Tree Construction

The observed plant species observed in the plots were standardized using the plant list (<www.theplantlist.org>) for botanical nomenclature. Subsequently, a species‐level phylogenetic tree was constructed using the “V. PhyloMaker” package in R (Jin and Qian 2019; Smith and Brown 2018), which includes 74,533 species of extant vascular plants (Jin and Qian 2019). Furthermore, the Interaction Tree Of Life (iTOL) platform was used to visualize a phylogenetic tree.

#### 2.3.2 Taxonomic and Phylogenetic Diversity

Taxonomic diversity was assessed using three metrics: species richness, Shannon–Wiener index, and Pielou evenness index, with the following formulas:

(1) Richness:R=S

(2) $$\text{Shannon}–\text{Wiener:}\;H^{\prime }=-\sum_{i=1}^{s}\frac{N_{i}}{N}\log_{2}\frac{N_{i}}{N}$$

(3) $$\text{Pielou:}\;E=\frac{H^{\prime }}{\mathrm{lns}}$$

where *S* is the number of species in the community, *N*_*i*_ is the number of species *i* in the community, and *N* is the total number of individuals of all species.

The phylogenetic diversity (*PD*) index was calculated as the sum of branch lengths along the nearest paths connecting all species on the phylogenetic tree (Faith 1992).

(4) $$\mathrm{PD}=\sum_{\left\{c\in \left.C\right\}\right.}L_{c}$$

In the above formula, *C* denotes the sum of all branches on the nearest path connecting all species on the phylogenetic tree, *c* represents a segment of the connecting node, and *L*_*c*_ is the length of segment *c*.

#### 2.3.3 Phylogenetic Structure of Plant Community

To measure the degree of phylogenetic relatedness among co‐occurring species within each community, the net relatedness index (*NRI*) and nearest taxon index (*NTI*) were utilized to describe the phylogenetic structure of the community (Webb et al. 2008).

(5) $$\mathrm{NRI}=-1\times \frac{{\mathrm{MPD}}_{S}-{\mathrm{MPD}}_{r}}{\mathrm{SD}\left({\mathrm{MPD}}_{r}\right)}$$

(6) $$\mathrm{NTI}=-1\times \frac{{\text{MNTD}}_{S}-{\text{MNTD}}_{r}}{\mathrm{SD}\left({\text{MNTD}}_{r}\right)}$$

where *MPDs* represents the mean phylogenetic distance among all species in the plot, while *MPDr* represents the standardized mean phylogenetic distance under a random distribution model. The random model was constructed by randomly permuting the tips of the phylogenetic tree for 999 times. The *SD* (*MPDr*) denotes the standard deviation of *MPDr*. Similarly, *MNTDs* represents the average phylogenetic distance between species in the community and their nearest phylogenetic neighbor. *MNTDr* represents the standardized average nearest phylogenetic distance under the random distribution model, with *SD* (*MNTD*_*r*_) as the standard deviation of *MNTDr*.

When both *NRI* and *NTI* are positive (> 0), species within a community are more closely related than expected under a null model, indicating phylogenetic clustering driven by environmental filtering. Conversely, when both are negative (< 0), species are more distantly related than expected, reflecting phylogenetic dispersion due to competitive exclusion or niche differentiation. When *NRI* and *NTI* approximate zero, community assembly reflects random processes, such as stochastic colonization or dispersal limitation (Jin and Qian 2019).

### 2.4 Environmental Variables

To quantify the main shape attributes of soil patches, we measured three key metrics: patch area (*PA*, m^2^), perimeter (*PM*, m), and fractal dimension (*PFD*). The fractal dimension (*PFD*) was calculated using the formula *PFD* = 2 * (ln(*PM*/4)/ln(*PA*)), where *PM* is the patch perimeter (m) and *PA* is the patch area (m^2^) (McGarigal et al. 2012; Zhang et al. 2017). This formula, based on the perimeter–area relationship, quantifies the irregularity and complexity of patch boundaries, as commonly applied in landscape ecology to link patch geometry to biodiversity and resource distribution (Zhang et al. 2017).

The mean values of soil depth (*Sd*, cm), moisture (*SWC*, %), total carbon (*TC*, g/kg), and total nitrogen (*TN*, g/kg) were used to assess the resource availability of soil patches (Bangroo et al. 2020; Liu et al. 2015). In addition, the coefficient of variation (*CV*) for soil depth, water content, total carbon, and total nitrogen was calculated as *CV* = *SD*/*MEAN* to evaluate the resource heterogeneity of soil patches, where *SD* is the standard deviation, *MEAN* is the mean value (Bangroo et al. 2020; Baskan et al. 2013; Zhang et al. 2014). Coefficient of variation (*CV*) thresholds were defined as strong (≥ 36%), moderate (16%–35%), or weak (≤ 15%) variation (Guan et al. 2012).

To assess soil depth availability and heterogeneity across different patches, measurements were conducted at points where soil depth exceeded 0 cm within each patch. Soil depth availability was represented by the mean of the measured soil depth, while soil depth heterogeneity was evaluated using the coefficient of variation (*CV*). Similar approaches were employed to evaluate the availability and heterogeneity of other soil properties, including soil moisture, total carbon, total nitrogen, and the carbon‐to‐nitrogen ratio, in various soil patches.

### 2.5 Statistical Analysis

Frequency distributions of patch area, soil depth, moisture, and nutrient content were visualized using Origin 2021. Additionally, the top 10 most frequent plant families and genera were also plotted. To investigate the relationships between patch properties, resource availability, resource heterogeneity, and diversity indices, Pearson correlation analysis was conducted using the “ggcor” R package. The relative importance of environmental factors contributing to species and phylogenetic diversity was determined using a random forest model implemented with the R package “randomForest”. In addition, linear mixed‐effects models (LMM), with site as a random effect and all environmental variables as fixed effects, were fitted using the R package “lme4” to evaluate the effect sizes and directions on species and phylogenetic diversity. Finally, variance decomposition analysis was performed using the R package “vegan” to quantify the relative contributions of patch properties, resource availability, and resource heterogeneity to species and phylogenetic diversity patterns. All statistical analyses were conducted in R 4.2.3.

## 3 Results

### 3.1 Soil Patch Properties, Diversity, and Species Composition

A total of 43 soil patches were investigated across 27 large plots (10 m × 10 m). The soil patch areas ranged from 0.15 to 67.19 m^2^, with an average area of 13.68 m^2^. Small patches (0–5 m^2^ and 5–10 m^2^) predominated in the karst region, comprising half of the total patches, while large patches (> 25 m^2^) were only six (Figure S2). The patch area (*PA*) varied from 0.15 to 67.19 m^2^, patch perimeter (*PM*) varied from 1.54 to 98.56 m, and fractal dimensions (*PFD*) ranged from 0.09 to 2.80 (Table 1).

**TABLE 1:** Statistical analysis of soil patch properties during the early restoration stage of karst abandoned lands.

Variable	Min	Max	Mean	SD	CV
Patch area (*PA*)	0.15	67.19	13.68	12.94	0.95
Patch perimeter (*PM*)	1.54	98.56	26.64	17.72	0.67
Fractal dimension (*PFD*)	0.09	2.80	1.57	0.41	0.26

In addition, species richness (*R*) exhibited a broad range from 3 to 41 species, with an average of 16.39 and a standard deviation of 8.31, indicating significant variation in species count across patches (Table 2). Similarly, the Shannon–Wiener diversity index (*H*′) also showed considerable variation, ranging from 0.39 to 3.76, with a mean of 2.76 and an *SD* of 0.74, reflecting differences in species diversity. In addition, phylogenetic diversity (*PD*) spanned a wide range from 636.84 to 3162.35, averaging at 1707.10 with an *SD* of 607.98. In terms of phylogenetic structure, the net relatedness index (*NRI*) varied from −0.88 to 1.69, averaging at 0.52 with an *SD* of 0.69. The nearest taxon index (*NTI*), another indicator of phylogenetic structure, ranged from −0.61 to 1.27, with a mean of 0.45 and an *SD* of 0.34 (Table 2).

**TABLE 2:** Plant species, phylogenetic diversity, and phylogenetic structure during the early restoration stage of karst abandoned lands.

Variable	Min	Max	Mean	SD
Species richness (*R*)	3.00	41.00	16.39	8.31
Shannon–Wiener (*H′*)	0.39	3.76	2.76	0.74
Pielou index (*E*)	0.36	1.34	1.03	0.18
Phylogenetic diversity (*PD*)	636.84	3162.35	1707.10	607.98
Net relatedness index (*NRI*)	−0.88	1.69	0.52	0.69
Nearest taxon index (*NTI*)	−0.61	1.27	0.45	0.34

### 3.2 Soil Resource Availability and Resource Heterogeneity Across Soil Patches

**FIGURE 2:**
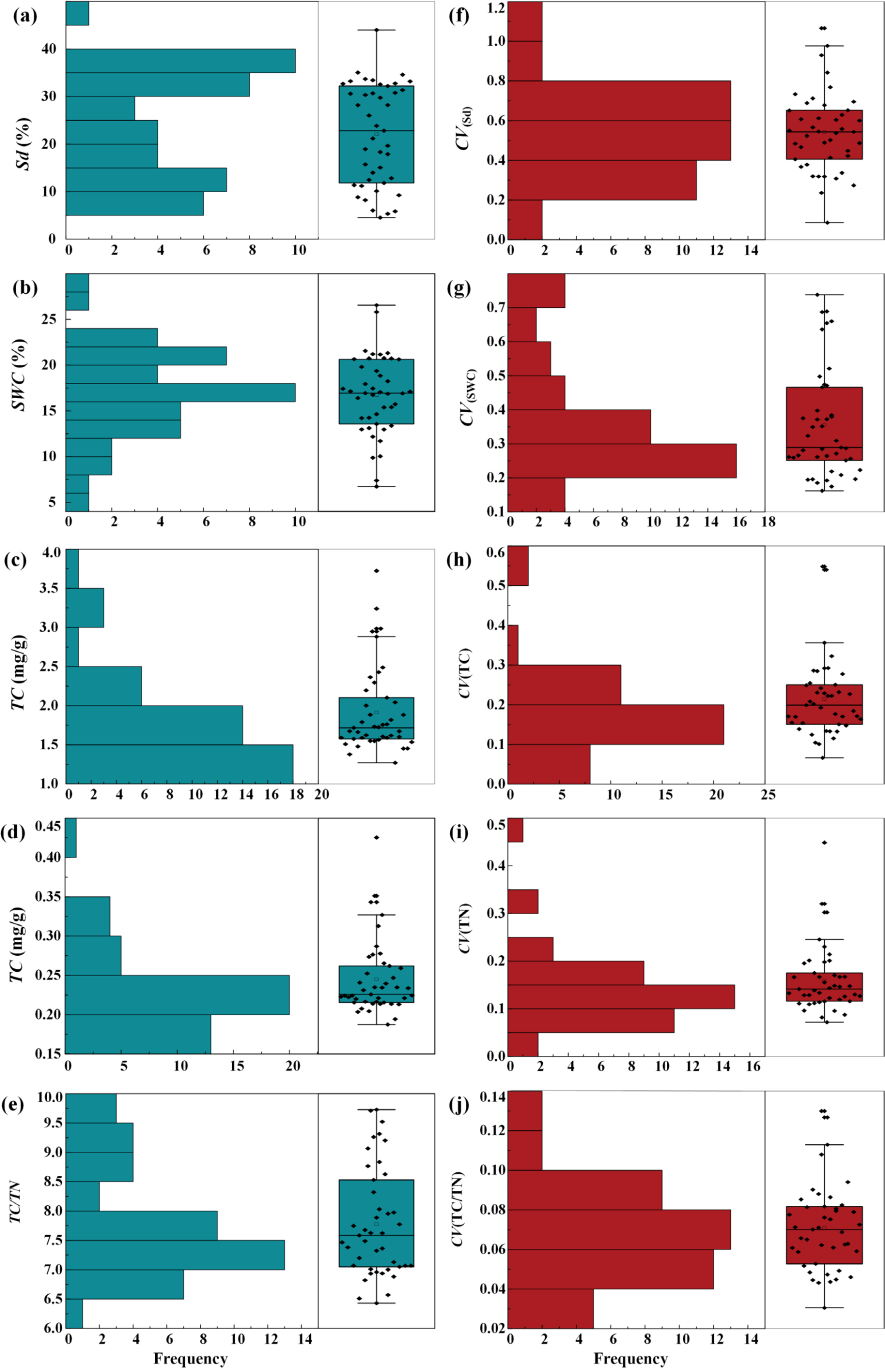
The frequency distribution and average value of the parameter of soil resource availability (a–e) and resource heterogeneity (f–j) in 43 soil patches. Sd, soil depth; SOC, soil water content; TC, soil total carbon content; TN, soil total nitrogen content; TC/TN, carbon‐to‐nitrogen ratio.

Soil resource availability was assessed through the mean values of soil depth, water content, total carbon content, total nitrogen content, and the carbon‐to‐nitrogen ratio (Figure 2a–e). Mean soil depth (*Sd*) was 24.27 cm, primarily ranging from 5 to 15 cm and 30 to 40 cm. Soil water content (*SWC*) averaged 17.17%, with the most frequent values ranging between 12.50% and 22.50%. Total carbon content (TC) averaged 1.82 mg/g, mainly ranging from 1.00 to 2.00 mg/g. Similarly, the average soil total nitrogen content (*TN*) was 0.23 g/mg, primarily within the range of 0.15 to 0.25 mg/g. The carbon‐to‐nitrogen ratio (*TC*: *TN*) averaged 7.78, with the most values distributed between 6.50 and 8.00 (Figure 2a–e).

Regarding resource heterogeneity, both soil depth and soil moisture content displayed the highest heterogeneity, with mean coefficients of variation (*CV*) of 0.53 and 0.37, respectively. Soil total carbon content (*TC*) showed moderate variation with an average *CV* of 0.18. Conversely, soil total nitrogen content (*TN*) and the carbon‐to‐nitrogen ratio (*TC*/*TN*) displayed low variability, with mean *CV*s of 0.14 and 0.07, respectively (Figure 2f–j).

### 3.3 Relationship Between Soil Patch Properties, Resource Availability, Resource Heterogeneity, and Biodiversity

**FIGURE 3:**
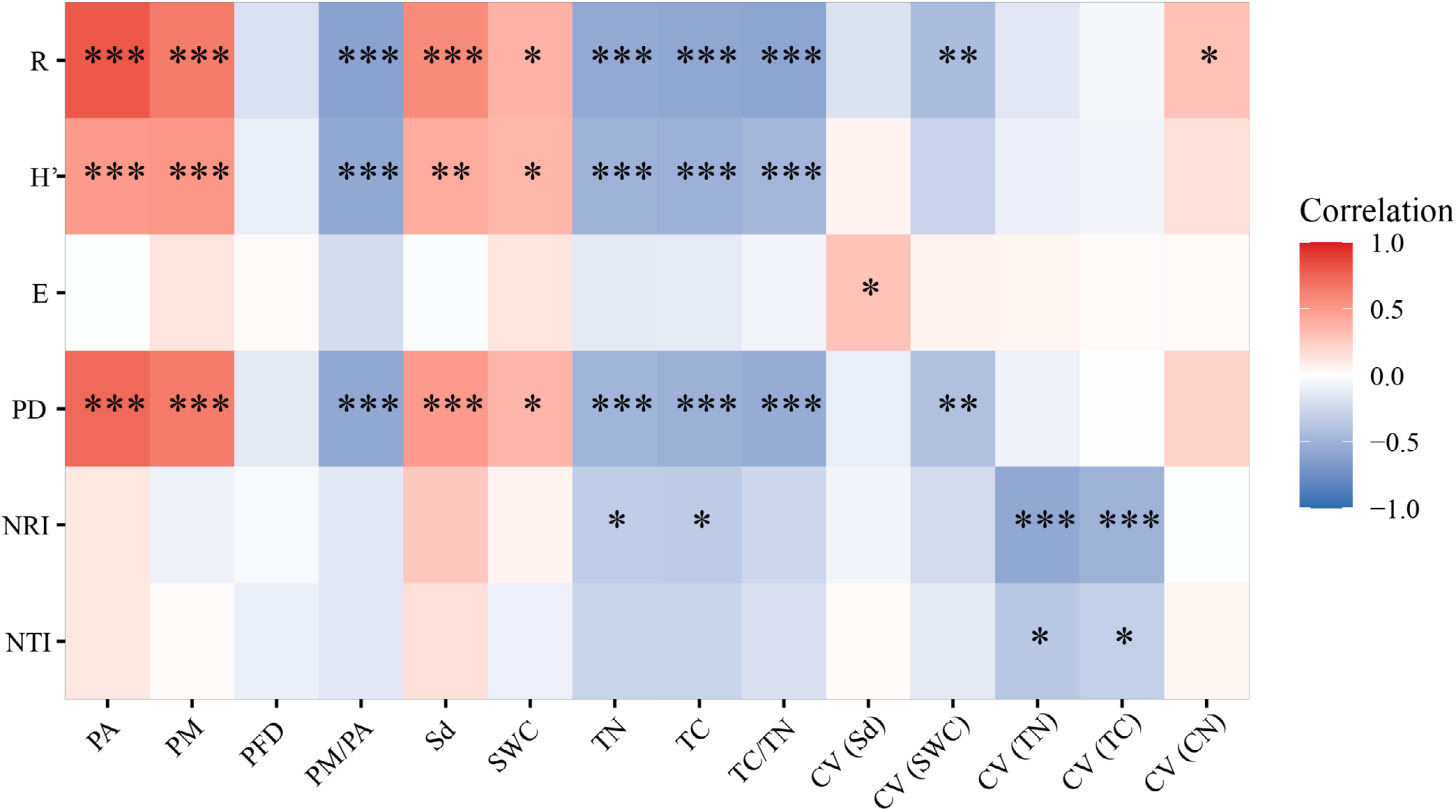
Correlation analysis between patch properties, resource availability, resource heterogeneity and biodiversity. *CV*_(Sd)_, the coefficient of variation of soil depth; *CV*_(SWC)_, the coefficient of variation of soil water content; *CV*_(TC)_, the coefficient of variation of soil total carbon content; *CV*_(TC/TN)_, the coefficient of variation of soil carbon‐to‐nitrogen ratio; *CV*_(TN)_, the coefficient of variation of soil total nitrogen content; *E*, Pielou index; *H′*, Shannon–Wiener index; *NRI*, net relatedness index; *NTI*, nearest taxon index; *P/A*, perimeter‐ to‐ area ratio; *PD*, phylogenetic diversity; *R*, species richness; Sd, soil depth; SWC, soil water content; TC, soil total carbon content; TC/TN, carbon‐to‐nitrogen ratio; TN, soil total nitrogen content. Red and blue indicate positive and negative correlations between leaf functional traits, respectively.

In terms of soil patch properties, both patch area and perimeter exhibited significant positive effects on species richness (*R*), Shannon–Wiener index (*H*′), and phylogenetic diversity (*PD*), whereas fractal dimension did not significantly affect plant diversity (Figure 3). Regarding resource availability indices, the mean values of soil depth (*Sd*) and water content (*SWC*) significantly positively correlated with *R*, *H*′, and *PD* (Figure 3). Conversely, the mean values of soil total carbon (*TC*), total nitrogen (TN), and carbon‐to‐nitrogen ratio (*TC/TN*) showed significant negative correlations with *R*, *H*′, and *PD*. In addition, within the resource heterogeneity indices, only the coefficient of variation (*CV*) for soil water content (*SWC*) was significantly negatively correlated with *R* and *PD*. Moreover, soil depth heterogeneity was the only shape characteristic index that showed a significant positive influence on species evenness (*E*) (Figure 3). In terms of the phylogenetic structure, the net relatedness index (*NRI*) showed a significant negative correlation with the mean values and *CV* of *TC* and *TN*. Furthermore, the nearest taxon index (*NTI*) only exhibited a significant negative correlation with the *CV* of *TC* and *TN* (Figure 3).

### 3.4 Relative Importance of Soil Patch Properties, Resource Availability, and Resource Heterogeneity on Biodiversity

**FIGURE 4:**
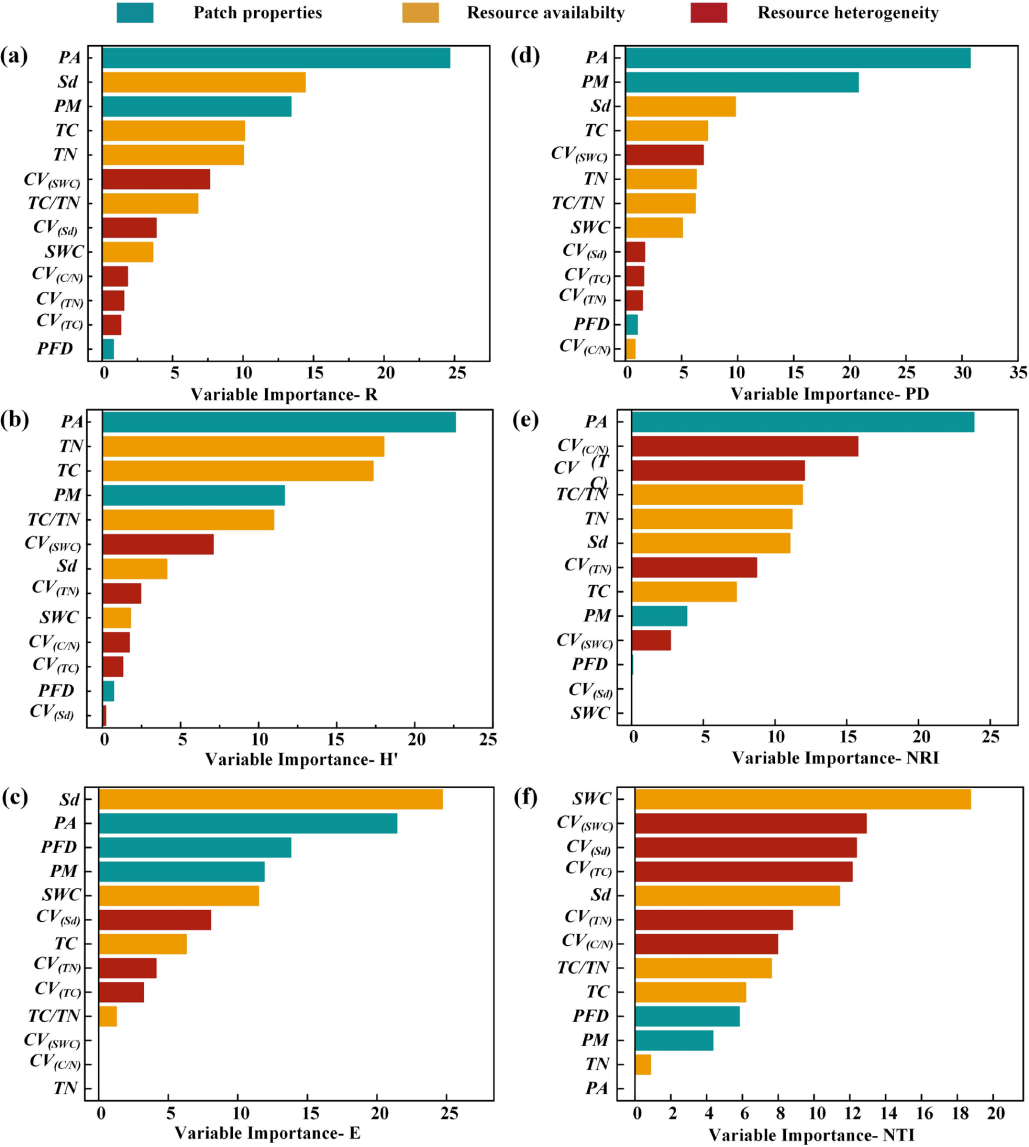
Assessment of the importance of each environmental factor for *R* (a), *H′* (b), *E* (c), *PD* (d), *NRI* (e), and *NTI* (f). The different colors of the bars signify the different predictor factors from patch properties, resource availability, and resource heterogeneity. Species richness (*R*), Shannon–Wiener index (*H′*), phylogenetic diversity (*PD*), net relatedness index (*NRI*) and nearest taxon index (*NTI*).

Random forest model showed that both soil patch properties and resource availability played crucial roles in species diversity indices and phylogenetic diversity indices in karst natural recovery areas. Patch area (*A*) emerged as the most critical factor influencing species richness (*R*, 24.9%), Shannon–Wiener (*H*′, 22.5%), phylogenetic diversity (*PD*, 30%), and *NRI* (19%), respectively (Figure 4). Notably, soil depth was identified as the most influential factor affecting species evenness (*E*, 25%) (Figure 4). Resource heterogeneity (e.g., *TC* and *TN*) had a relatively minor effect on species diversity but exerted a stronger influence on phylogenetic diversity. Among the resource heterogeneity indices, the *CV*s of the carbon‐to‐nitrogen ratio (*C*/*N*) and total carbon (*TC*) were the primary factors impacting the *NRI*. Furthermore, the *CV*s of soil water content (*SWC*), soil depth (*Sd*), and *TC* were identified as key factors influencing the *NTI* (Figure 4).

**FIGURE 5:**
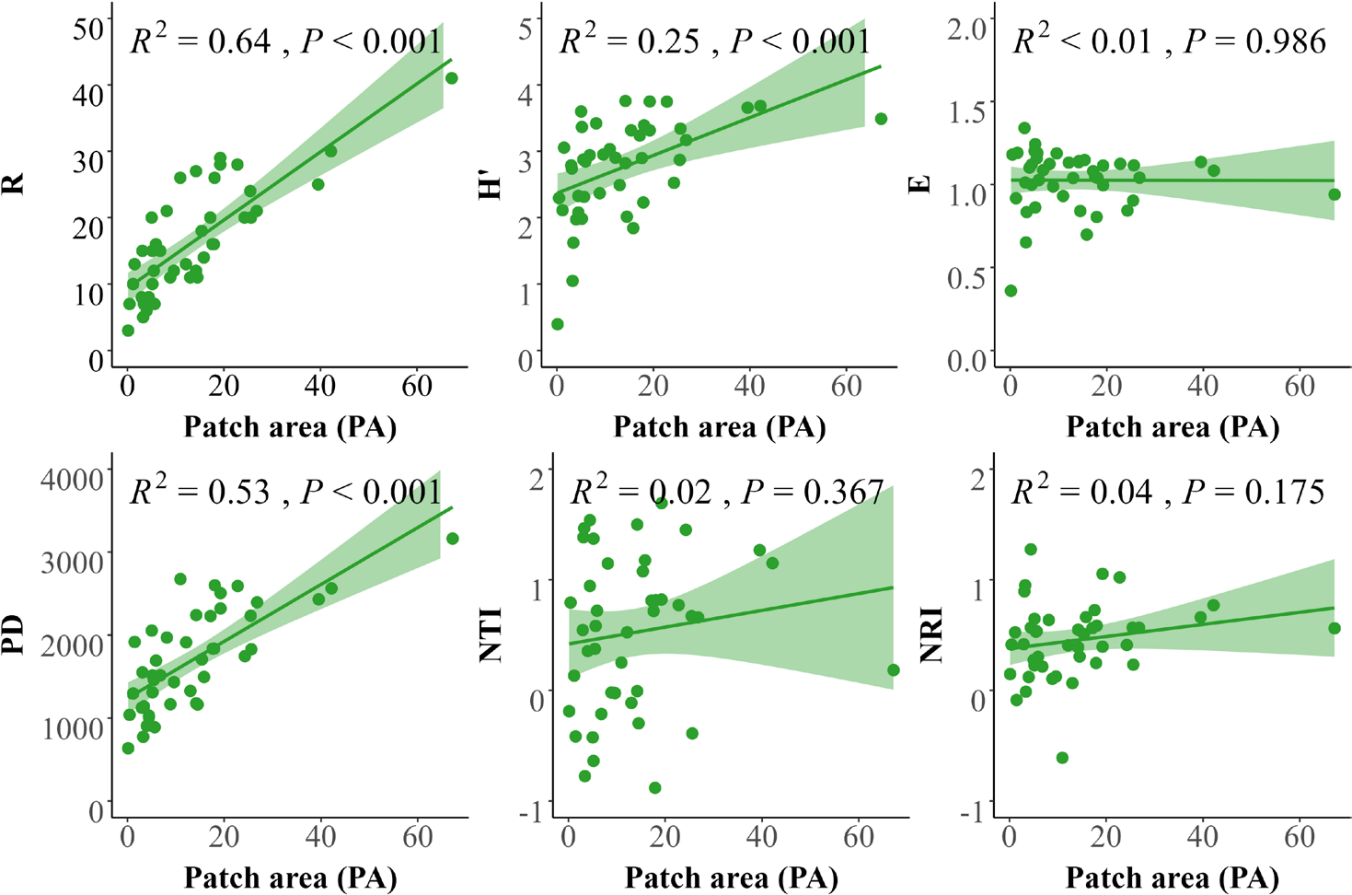
Relationships between patch area (PA) and plant species diversity, phylogenetic diversity, and phylogenetic structure during the early restoration stage of karst abandoned lands. Species richness (*R*), Shannon–Wiener index (*H*′), phylogenetic diversity (*PD*), net relatedness index (*NRI*), and nearest taxon index (*NTI*).

Furthermore, plant diversity consistently increased with soil patch area (*p* < 0.001; Figure 5). For species diversity, there was a stronger positive relationship with patch area (*R* ^2^ = 0.64, *p* < 0.001) than Shannon–Wiener diversity (*R* ^2^ = 0.25, *p* < 0.001) (Figure 5). Similarly, phylogenetic diversity (*PD*) also increased significantly with patch area (*R* ^2^ = 0.53, *p* < 0.001). However, patch area (*A*) had no significant effect on phylogenetic structure (*NRI* and *NTI*; *p* > 0.05). Most herbaceous communities exhibited positive *NRI* and *NTI* values (particularly *NRI* > 0), indicating phylogenetic clustering driven by deterministic processes such as environmental filtering. However, *NRI* and *NTI* values ranged between −1.96 and 1.96, suggesting that random processes also contribute to community assembly (Figure 5).

### 3.5 Effects of Soil Patch Properties, Resource Availability, and Resource Heterogeneity on Biodiversity

**FIGURE 6:**
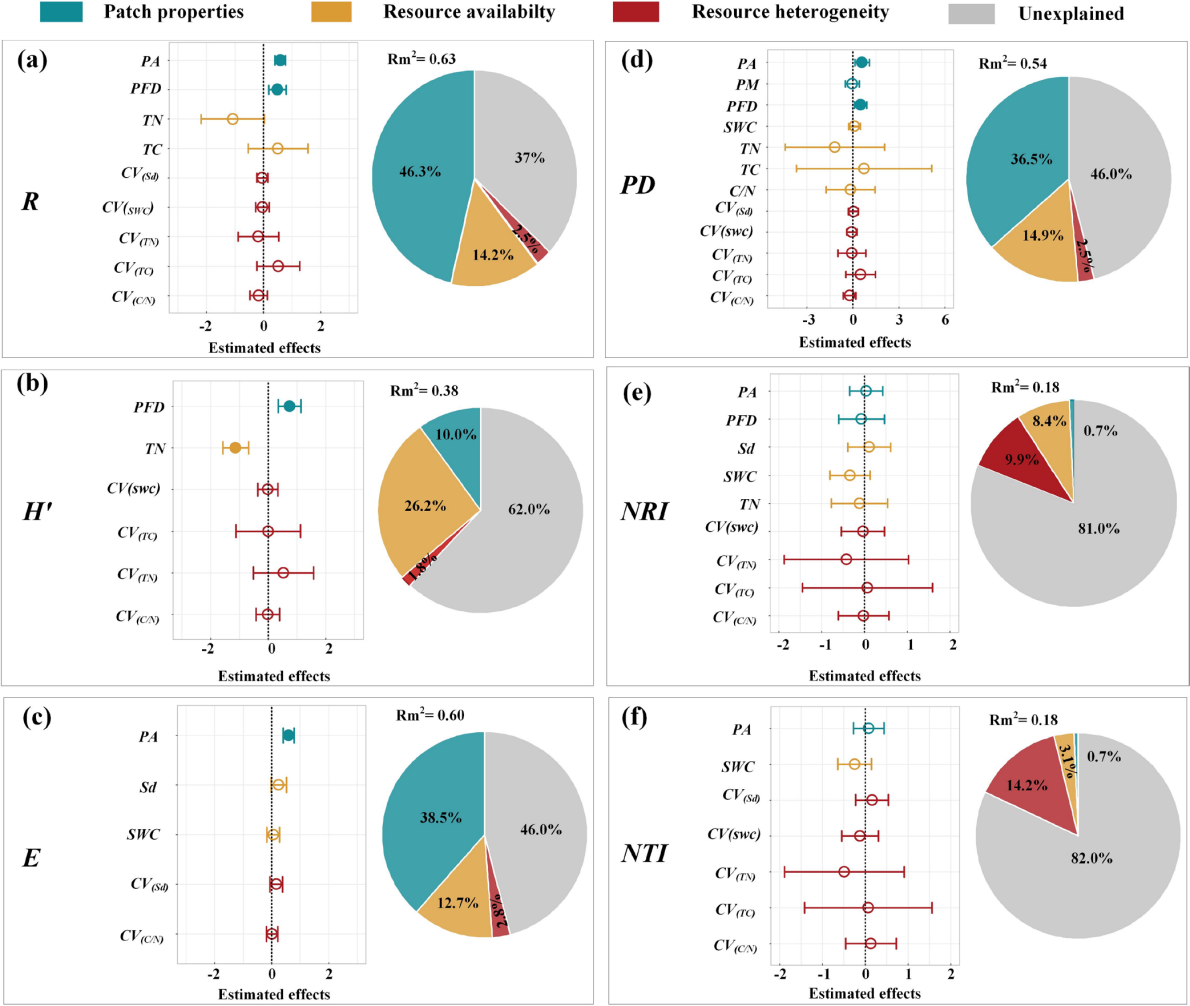
Effects of environmental factors on species richness (*R*, a), Shannon–Wiener diversity (*H*′, b), species evenness (*E*, c), Phylogenetic diversity (*PD*, d), net relatedness index (*NRI*, e), and nearest taxon index (*NTI*, f). Solid circles and lines represent standardized parameter estimates and their 95% confidence intervals, with solid circles indicating significant effects (*p* < 0.05) and hollow circles indicating nonsignificant effects (*p* > 0.05). Pie charts show the relative importance of each variable in explaining variance.

The linear mixed‐effects model showed that soil patch properties had a strong explanatory power on species diversity indices, accounting for 46.3% of the variation in species richness (*R*), 10.0% in the Shannon–Wiener index (*H*′), and 38.3% in species evenness (*E*) (Figure 6a–c). Patch area (*PA*) had a significant positive effect on both *R* and *E* (Figure 6). In contrast, resource availability (26.2%) had the greatest impact on *H*′, with total nitrogen content showing a significant negative effect on *H*′. In addition, patch properties, especially patch area, displayed the highest explanatory power (36.5%) on the *PD* than resource availability (14.9%) and heterogeneity (2.5%), showing a significant positive effect (Figure 6). For phylogenetic structure, while resource availability and resource heterogeneity had relatively higher explanatory power for *NRI* and *NTI*, their effects were not statistically significant (Figure 6).

**FIGURE 7:**
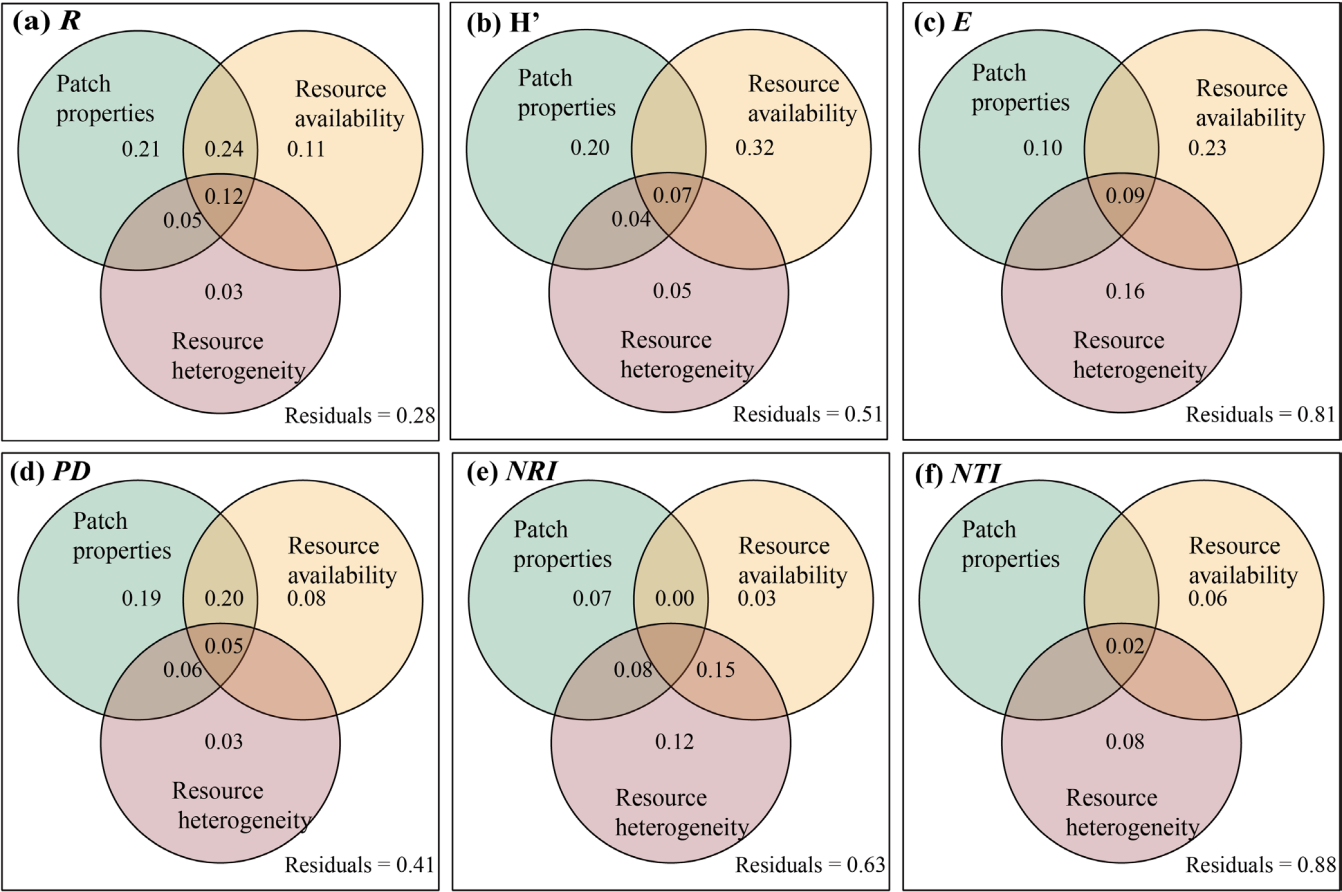
Variation partitioning analysis of patch properties, resource availability, and resource heterogeneity to *R* (a), *H′* (b), *E* (c), *PD* (d), *NRI* (e) and *NTI* (f). Species richness (*R*), Shannon–Wiener index (*H′*), phylogenetic diversity (*PD*), net relatedness index (*NRI*) and nearest taxon index (*NTI*).

Furthermore, the variance decomposition model revealed that the combined effects of soil patch properties, soil resource availability, and resource heterogeneity accounted for 72% of the total variation in *R*, 49% in *H*′, 59% in *PD*, and 37% in *NRI*, respectively (Figure 7). Resource availability explained a higher proportion of variation in *H*′ and *E* compared to resource heterogeneity (Figure 7a–c). Notably, in terms of *R* and *PD*, soil patch properties exhibited a higher explanatory power than resource availability (Figure 7).

## 4 Discussion

### 4.1 Patch Area Is the Most Influential Factor in Shaping Species Diversity and Phylogenetic Diversity

The dimension of patch area is fundamental in defining patch properties and serves as a pivotal driver of biodiversity (Ylisirniö et al. 2016). Numerous studies have demonstrated that species richness tends to increase with patch area across various ecosystems (Resetarits et al. 2022; Storch et al. 2012; Zhang, Zhang, et al. 2021). Consistent with these findings, our results revealed that larger patch areas exhibited a significant positive effect on species richness, Shannon–Wiener index, and phylogenetic diversity (Figure 4), which was further supported by our random forest and linear regression analysis (Figures 4 and 5). These findings highlight the dominant role of patch area in shaping both species and phylogenetic diversity patterns at our study site, though its relative importance may vary in other karst regions with different elevation gradients, soil properties, or land‐use histories (Wu et al. 2025; Zhang, Chen, et al. 2021). This pattern aligns with island biogeography theory, which posits that larger habitat patches, analogous to islands, support higher species richness due to increased immigration rates and reduced extinction risks (Warren et al. 2015; Whittaker et al. 2008). In karst ecosystems, larger patches likely enhance colonization by providing more stable resources and connectivity, while smaller patches, like smaller islands, limit species persistence due to constrained habitat area and resource availability (Losos and Ricklefs 2009). Several mechanisms may explain this relationship. First, in rocky desertification areas, soil depth represents a critical aspect of soil resource availability (Price et al. 2017). Specifically, larger patch areas in karst landscapes typically have greater soil depths and volume, increasing resource availability for plant growth (Liu et al. 2020). Secondly, small populations in smaller soil patches face heightened extinction risks, even with ongoing colonization efforts, due to genetic constraints and limited colonization success (Alzate et al. 2019). Thirdly, larger patches often encompass diverse microhabitats, thus offering more ecological niches that promote species coexistence, as predicted by niche theory (Carroll et al. 2011). However, in other karst ecosystems, such as those influenced by varying soil depth gradients, patch shape and connectivity may play a larger role than area due to differing dispersal dynamics and land‐use histories (Liu et al. 2024). These mechanisms collectively underscore patch area's dominant role in shaping biodiversity patterns at our study site in karst ecosystems.

### 4.2 Soil Resource Availability Has a Greater Effect on Species Diversity Than Resource Heterogeneity

Our findings highlighted that soil depth was the second most important factor influencing species richness after patch area (Figure 5a). This observation aligns with a recent study indicating that the resource availability of vegetation growth space plays a crucial role in influencing species diversity in karst regions (Liu et al. 2020). Soil serves as the foundation for plant survival and growth, supplying essential resources such as water and nutrients. During the early restoration stage of karst abandoned lands, we noted that pioneer species from Asteraceae, Poaceae, and Crassulaceae families dominated, owing to their remarkable ability to endure low fertility and drought conditions. Notably, species such as *Carex* spp., *Artemisia scoparia* , and *Elymus sibiricus* thrive due to well‐adapted root systems suited to shallow soils (Table S1). In addition, the observed rise in soil depth (10–40 cm in this study) enhanced soil volume, water content, and nutrient availability. This facilitated a more favorable environment for the colonization and survival of diverse species in this region (Figure 2).

Furthermore, our results confirmed that soil resource availability has a greater impact on species diversity than soil resource heterogeneity during the early restoration stage in abandoned rocky lands (Figures 4 and 6). This finding is consistent with a recent study that soil resource availability is more influential than heterogeneity in shaping species diversity within karst ecosystems (Liu et al. 2021). Moreover, this pattern has also been observed across various other ecosystems (Bartels and Chen 2010; El‐Keblawy 2017; Helbach et al. 2022; Wilsey et al. 2005). For instance, in other karst regions of Southwest China, soil depth and water availability similarly drive species richness, but nutrient availability (e.g., phosphorus) may be more limiting in regions with different lithology, such as dolomite‐dominated karst, or under varying restoration stages (Zhang et al. 2015; Tong et al. 2017). Notably, rocky desertification areas are characterized by exposed bedrock, slow soil formation, rapid soil erosion, and limited soil water and nutrient capacity, collectively posing potential limitations on plant growth (Zhang et al. 2019). Consequently, while smaller patches may display greater soil heterogeneity, plant roots frequently extend beyond these patches, increasing the risk of plant mortality in unfavorable segments (Laanisto et al. 2013). The limited availability of resources, exacerbated by soil heterogeneity, further hinders plant survival and growth, consequently restricting species diversity in abandoned karst farmlands (Gazol et al. 2013; Liu et al. 2021).

### 4.3 Soil Resource Availability and Heterogeneity Differentially Shape Phylogenetic Diversity and Community Assembly

Our findings revealed that soil resource availability had a greater impact on phylogenetic diversity (*PD*), with mean soil depth and water content (*SWC*) positively correlated with *PD* (Figure 3), reflecting their role in alleviating resource constraints (Liu et al. 2020; Price et al. 2017). Conversely, mean soil total carbon (*TC*), total nitrogen (*TN*), and carbon‐to‐nitrogen ratio (*TC*/*TN*) were negatively correlated with *PD*, suggesting that high nutrient levels may favor dominant pioneer species, such as Asteraceae and Poaceae, reducing the evolutionary breadth of communities during early restoration (Campos et al. 2022; Zhang et al. 2019). Conversely, soil resource heterogeneity, notably the coefficient of variation (*CV*) of *SWC*, also influenced *PD*, with a significant negative correlation indicating that high water variability restricts diverse lineages, favoring species adapted to stable moisture conditions (Deák et al. 2020). This differential impact of heterogeneity on species versus phylogenetic diversity can be attributed to environmental filtering and niche conservatism. High soil resource heterogeneity, particularly in SWC, acts as a strong filter in karst ecosystems, selecting for species with shared ecological traits (e.g., drought tolerance), which are often phylogenetically clustered due to conserved adaptations within lineages (Cavender‐Bares et al. 2009; Webb et al. 2002). This reduces phylogenetic diversity by limiting the establishment of distantly related species. In contrast, species diversity is less affected by heterogeneity because resource availability, such as sufficient mean SWC or soil depth, supports a broader range of species regardless of phylogenetic relatedness, as long as they can tolerate the karst environment (Swenson and Enquist 2009). Regarding phylogenetic structure, the net relatedness index (*NRI*) showed significant negative correlations with both mean values and *CV* of *TC* and *TN* (Figure 3), suggesting phylogenetic dispersion driven by nutrient availability and variability, as high or variable nutrient levels enable distantly related species to coexist by reducing niche overlap (Cadotte et al. 2012; Gastauer et al. 2021).

For community assembly, we observed that herbaceous communities exhibited positive *NRI* and *NTI* values, particularly *NRI* > 0, indicating phylogenetic clustering driven by environmental filtering under resource scarcity (Webb et al. 2002). However, *NRI* and *NTI* values ranging between −1.96 and 1.96 reveal that random processes, such as stochastic colonization and priority effects, also contribute to community assembly, especially in highly heterogeneous patches where variable resources limit deterministic filtering (Hubbell Stephen 2001; Kraft et al. 2007). In nitrogen‐limited karst ecosystems, high soil heterogeneity exacerbates niche overlap in smaller patches, restricting coexistence of closely related species, while moderate heterogeneity fosters phylogenetic dispersion by creating diverse microhabitats that support distantly related species (Gastauer et al. 2021). These dynamics highlight a complex interplay of deterministic and stochastic processes, with resource availability driving *PD* through filtering and heterogeneity modulating assembly via niche differentiation and stochasticity (Cavender‐Bares et al. 2009; Cornwell and Ackerly 2009; Kraft and Ackerly 2010; Swenson 2011). These processes, observed at our study site, may vary in karst regions with different climatic or lithological conditions, where, for example, phosphorus limitation or vegetation succession stages may dominate filtering dynamics (Tong et al. 2017; Zhang et al. 2015). Restoration strategies should enhance soil depth and stabilize water availability to support diverse lineages, while managing heterogeneity to balance deterministic and stochastic assembly for stable karst communities (Perring et al. 2015).

### 4.4 Comparison With Non‐Karst Restoration Communities

Our findings on the dominant role of patch area and soil resource availability in karst ecosystems share similarities with restoration dynamics in non‐karst communities, such as temperate grasslands and abandoned agricultural lands, where larger patch areas consistently enhance species richness by providing greater resource availability and niche diversity (Wilsey et al. 2005; Bartels and Chen 2010). However, the pronounced influence of soil depth in our study is more specific to karst ecosystems, where shallow soils and exposed bedrock limit resource availability compared to deeper, more fertile soils in non‐karst systems (Török et al. 2011). Additionally, while soil resource availability outweighs heterogeneity in shaping species diversity in both karst and non‐karst contexts, the extreme edaphic constraints in karst regions amplify the importance of water and nutrient availability, leading to stronger environmental filtering of pioneer species (Yan et al. 2023). These comparisons suggest that while general ecological principles apply across ecosystems, karst‐specific constraints necessitate tailored restoration strategies.

### 4.5 Implications and Limitations

This study innovatively examines the roles of patch properties, soil resource availability, and heterogeneity from a patch‐scale perspective, revealing their impacts on species diversity, phylogenetic diversity, and community assembly in early restoration of karst abandoned lands, with environmental filtering and stochastic processes shaping herbaceous communities (Gazol et al. 2013; Hubbell Stephen 2001). However, several limitations warrant consideration. First, our study, conducted at a single karst site in southwest China with single‐season soil measurements (*SWC*, *TC*, *TN*), may limit the generalizability of our findings to other karst regions with varying climates, lithologies, or land‐use histories, potentially overlooking temporal variability that influences resource dynamics and community assembly (Ye et al. 2022; Adler et al. 2011). For instance, seasonal shifts in soil moisture and nutrient availability could differentially affect species richness by favoring specific plant functional groups across seasons, underscoring the importance of multi‐seasonal sampling to capture these temporal effects (Hu et al. 2024). Second, our soil resource assessments were limited to *SWC*, *TC*, and *TN*, omitting measurements of soil phosphorus (*P*) and potassium (*K*), which constrain plant growth and diversity in karst ecosystems (Liu et al. 2018). For example, *P* availability often constrains plant growth and diversity in calcareous soils due to fixation with calcium, potentially interacting with observed heterogeneity to shape phylogenetic clustering or overdispersion (Lambers et al. 2008; Vitousek et al. 2010). Third, our focus on soil resource indices excluded microbial community dynamics, which likely mediate nutrient cycling and plant–soil interactions critical for karst restoration (Hu et al. 2022). Fourth, the lack of investigation into biotic interactions, such as competition, facilitation, or herbivory, which can mediate species richness and phylogenetic structure in heterogeneous environments (Callaway and Walker 1997; Brooker et al. 2008).

Despite these constraints, our patch‐scale approach advances understanding by elucidating how resource availability, including soil depth (*Sd*, cm), soil water content (*SWC*, %), total carbon (*TC*, g/kg), and total nitrogen (*TN*, g/kg), and heterogeneity (*CV* of *SWC*, *TC*, *TN*) drive phylogenetic diversity and community assembly, offering a novel framework beyond traditional landscape‐scale diversity metrics (Webb et al. 2002). Our work highlights how patch‐scale resource dynamics shape early restoration, providing a foundation for biodiversity conservation in fragile karst ecosystems. Future research should incorporate multi‐seasonal soil data, explicit land‐use history assessments, and microbial community analyses to enhance the robustness of patch‐scale insights and guide sustainable karst restoration.

## 5 Conclusions

Based on our single‐site study in a subtropical karst region, this study highlights the crucial roles of patch properties, soil resource availability, and soil resource heterogeneity in shaping species diversity, phylogenetic diversity, and phylogenetic structure during the early restoration of rocky desertification abandoned lands. Patch area, among the patch properties, emerged as the pivotal factor influencing both species diversity and phylogenetic diversity. Resource availability exerted a greater impact on species diversity than resource heterogeneity, with soil depth contributing positively to species richness by improving resource availability. Conversely, for phylogenetic structure (*NRI* and *NTI*), resource heterogeneity, especially variability in water content, had a more pronounced effect than resource availability. Furthermore, deterministic and stochastic processes jointly drove the community assembly of early herbaceous communities in karst abandoned farmland. In karst regions, while larger soil patches play a vital role in maintaining species diversity and ecosystem functions, smaller patches, despite their limited resource availability, should also be prioritized in conservation efforts through strategies like soil supplementation. Furthermore, natural restoration in karst abandoned land should be prioritized, and artificial restoration, such as single‐soil transfers and species transplants, should be minimized to maintain higher resource heterogeneity, which is vital for enhancing regional biodiversity.

## Author Contributions

**Weixue Luo:** conceptualization (equal), formal analysis (equal), methodology (equal), writing – review and editing (equal). **Ying Lei:** data curation (equal), formal analysis (equal), investigation (equal), writing – original draft (equal). **Yijie Zhao:** investigation (equal), writing – review and editing (equal). **Wenqi Yang:** investigation (equal), writing – review and editing (equal). **Haohan Du:** data curation (equal), investigation (equal). **Jie Luo:** investigation (equal), writing – review and editing (equal). **Xuman Guo:** resources (equal), writing – review and editing (equal). **Wenjing Tao:** methodology (equal), writing – review and editing (equal). **Zongfeng Li:** supervision (equal), writing – review and editing (equal). **Jianping Tao:** supervision (equal), writing – review and editing (equal). **Jinchun Liu:** conceptualization (equal), funding acquisition (equal), validation (equal), writing – review and editing (equal).

## Conflicts of Interest

The authors declare no conflicts of interest.

## Supporting information


**Appendix S1:** ece372417‐sup‐0001‐AppendixS1.docx.

## Data Availability

Data available via the Figshare Digital Repository: Luo (2025). https://doi.org/10.6084/m9.figshare.29431784.v1.
