# Supplementary material for: Patch Area and Soil Resource Availability Outweigh Heterogeneity in Shaping Karst Plant Diversity During Early Restoration
Source: Ecol Evol. 2025 Oct 30;15(11):e72417. doi: 10.1002/ece3.72417 (PMC12575285; doi:10.1002/ece3.72417)
Supplement: Supplementary file 1 — Appendix S1: ece372417‐sup‐0001‐AppendixS1.docx. [file ECE3-15-e72417-s001.docx]

**Supplementary information**

**Patch area and soil resource availability outweigh heterogeneity in shaping karst plant diversity during early restoration**

Weixue Luo ^1, 2^, Ying Lei ^1^, Yijie Zhao ^1^, Wenqi Yang ^1^, Haohan Du ^1^, Jie Luo ^1^, Xuman Guo ^1^, Wenjing Tao ^1^, Zongfeng Li ^1^, Jianping Tao ^1, 2^, Jinchun Liu ^1, 2*^

^1^ Key Laboratory of Eco-environments in Three Gorges Reservoir Region (Ministry of Education), Chongqing Key Laboratory of Plant Ecology and Resources Research in Three Gorges Reservoir Region, School of Life Sciences, Southwest University, Chongqing 400715, China

^2^ Chongqing Jinfo Mountain Karst Ecosystem National Observation and Research Station, Southwest University, Chongqing 400715, China

Email: W. Luo: [luowx0305@swu.edu.cn](mailto:luowx0305@swu.edu.cn);

Y. Lei: [651738106@qq.com](mailto:651738106@qq.com);

Y. Zhao: [3289383525@qq.com](mailto:3289383525@qq.com);

Q. Yang: [1466744379@qq.com](mailto:1466744379@qq.com);

H. Du: [wxrvynioppoi@qq.com](mailto:wxrvynioppoi@qq.com);

J. Luo: [rl_jie@163.com](mailto:rl_jie@163.com);

X. Guo: [861050694@qq.com](mailto:861050694@qq.com);

W. Tao: [taowj777@swu.edu.cn](mailto:taowj777@swu.edu.cn);

Z. Li: [lizfswu@swu.edu.cn](mailto:lizfswu@swu.edu.cn);

J. Tao: [taojp@swu.edu.cn](mailto:taojp@swu.edu.cn);

*Corresponding authors: E-mail: J. Liu: [jinchun@swu.edu.cn](mailto:jinchun@swu.edu.cn).

Present address: Southwest University, No.2 Tiansheng Road, Beibei District, Chongqing 400715, China

**This file includes:**

Number of pages:6; Number of Table: 2; Number of Figure: 2;

# Supplementary Materials

**Table S1** Importance values of species (top 5) in different patches

| Patch size | Species | Family | Importance value |
| --- | --- | --- | --- |
| 0-5 m^2^ | *Cayratia japonica* | Vitaceae | 0.35 |
|  | *Conyza canadensis* | Asteraceae | 0.32 |
|  | *Sedum sarmentosum* | Crassulaceae | 0.32 |
|  | *Artemisia annua* | Asteraceae | 0.21 |
|  | *Setaria viridis* | Gramineae | 0.19 |
|  |  |  |  |
| 5-10 m^2^ | *Conyza canadensis* | Asteraceae | 0.33 |
|  | *Sedum sarmentosum* | Crassulaceae | 0.32 |
|  | *Cayratia japonica* | Vitaceae | 0.28 |
|  | *Commelina communis* | Commelinaceae | 0.25 |
|  | *Oxalis corniculata* | Oxalidaceae | 0.15 |
|  |  |  |  |
| 10-15 m^2^ | *Conyza canadensis* | Asteraceae | 0.29 |
|  | *Oxalis corniculata* | Oxalidaceae | 0.29 |
|  | *Rubus idaeus* | Rosaceae | 0.24 |
|  | *Imperata cylindrica* | Gramineae | 0.23 |
|  | *Cayratia japonica* | Vitaceae | 0.19 |
|  |  |  |  |
| 15-20 m^2^ | *Conyza canadensis* | Asteraceae | 0.46 |
|  | *Artemisia argyi* | Asteraceae | 0.19 |
|  | *Sedum sarmentosum* | Crassulaceae | 0.18 |
|  | *Cynodon dactylon* | Gramineae | 0.16 |
|  | *Centella asiatica* | Apiaceae | 0.13 |
|  |  |  |  |
| 20-25 m^2^ | *Conyza canadensis* | Asteraceae | 0.41 |
|  | *Cynodon dactylon* | Gramineae | 0.26 |
|  | *Duchesnea indica* | Rosaceae | 0.23 |
|  | *Senecio scandens* | Asteraceae | 0.22 |
|  | *Rubus idaeus* | Rosaceae | 0.12 |
|  |  |  |  |
| 25-30 m^2^ | *Digitaria ciliaris* | Gramineae | 0.38 |
|  | *Conyza canadensis* | Asteraceae | 0.31 |
|  | *Oxalis corniculata* | Oxalidaceae | 0.20 |
|  | *Sedum sarmentosum* | Crassulaceae | 0.16 |
|  | *Commelina communis* | Commelinaceae | 0.15 |
|  |  |  |  |
| > 30 m^2^ | *Conyza canadensis* | Asteraceae | 0.30 |
|  | *Digitaria ciliaris* | Gramineae | 0.23 |
|  | *Sedum sarmentosum* | Crassulaceae | 0.22 |
|  | *Centella asiatica* | Apiaceae | 0.14 |
|  | *Bidens pilosa* | Asteraceae | 0.14 |


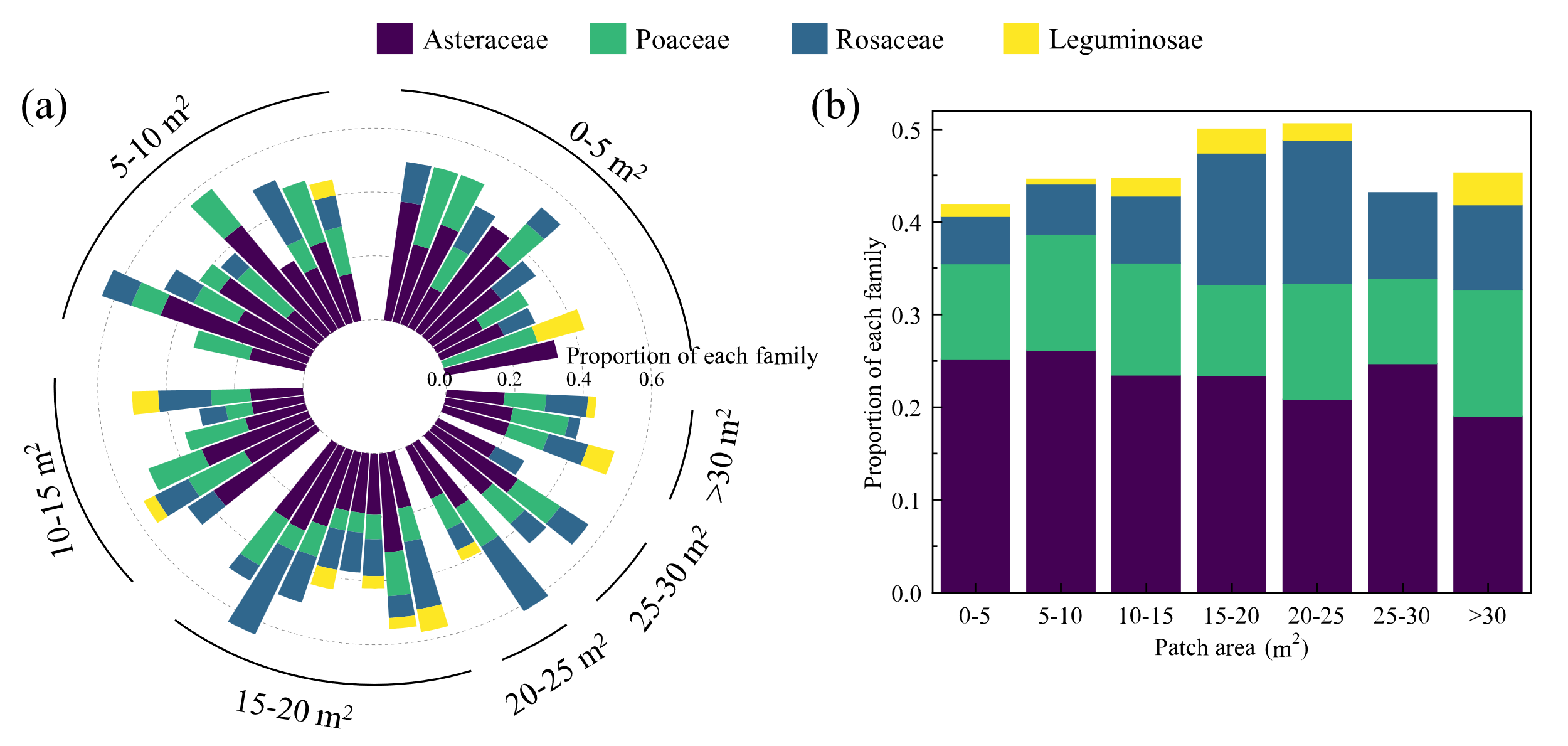


**Fig. S1** Percentage of each family under different patch sizes.

**Fig. S2** The frequency distribution of soil patch size during the early restoration stage of karst abandoned lands.
